# Personal protective measures and settings on the risk of SARS-COV-2 community transmission: a case–control study

**DOI:** 10.3389/fpubh.2023.1327082

**Published:** 2024-01-08

**Authors:** Aina Huguet-Torres, Enrique Castro-Sánchez, Laura Capitán-Moyano, Cristian Sánchez-Rodríguez, Miquel Bennasar-Veny, Aina M. Yáñez

**Affiliations:** ^1^Department of Nursing and Physiotherapy, University of Balearic Islands, Palma, Spain; ^2^Research Group on Global Health, University of Balearic Islands, Palma, Spain; ^3^College of Business, Arts, and Social Sciences, Brunel University London, Uxbridge, United Kingdom; ^4^Imperial College London, London, United Kingdom; ^5^Hospital Sant Joan de Déu, Palma, Spain; ^6^Research Group on Global Health and Lifestyle, Health Research Institute of the Balearic Islands (IdISBa), Palma, Spain; ^7^CIBER de Epidemiología y Salud Pública (CIBERESP), Carlos III Institute of Health (ISCIII), Madrid, Spain

**Keywords:** SARS-CoV-2, contact tracing, hand disinfection, physical distancing, masks, ventilation, respiratory tract diseases

## Abstract

**Background:**

During the SARS-CoV-2 pandemic, nurses of primary health care has been an important role in Spain. Even so, the data obtained in the tracing have been scarcely used to investigate the possible mechanisms of transmission. Few studies focused on community transmission, evaluating the effectiveness of individual protective measures and exposure environment. The main aim of the study was to evaluate the association between individual protective measures and SARS-CoV-2 transmission in the community and to compare secondary attack rates in different exposure settings.

**Methods:**

A case–control study from contact tracing of SARS-CoV-2 index patients. COVID-19 contact tracing was led by nurses at the COVID-19 Coordinating Centre in Majorca (Spain). During the systematic tracing, additional information for this study was collected from the index patient (social-demographic variables, symptoms, the number of close contacts). And also, the following variables from their close contacts: contact place, ventilation characteristics mask-wearing, type of mask, duration of contact, shortest distance, case-contact relationship, household members, and handwashing, the test result for SARS-CoV-2 diagnostic. Close contacts with a positive test for SARS-CoV-2 were classified as “cases” and those negative as “controls.”

**Results:**

A total of 1,778 close contacts from 463 index patients were identified. No significant differences were observed between the sexes but between age groups. Overall Secondary Attack Rate (SAR) was 24.0% (95% CI: 22.0–26.0%), 36.9% (95% CI: 33.2–40.6%) in closed spaces without ventilation and 50.7% (95% CI: 45.6–55.8%) in exposure time > 24 h. A total of 49.2% of infections occurred among household members. Multivariate logistic regression analysis showed that open-air setting (OR 0.43, 95% CI: 0.27–0.71), exposure for less than 1 h (OR 0.19, 95% CI: 0.11–0.32), and wearing a mask (OR 0.49, 95% CI: 0.28–0.85) had a protective effect transmission of SARS-CoV-2 in the community.

**Conclusion:**

Ventilation of the space, mask-wearing and shorter exposure time were associated with a lower risk of transmission in the community. The data obtained allowed an assessment of community transmission mechanisms and could have helped to improve and streamline tracing by identifying close contacts at higher risk.

## Introduction

The COVID-19 pandemic had an unprecedented impact on society, making it the pandemic with the greatest impact worldwide in recent times. SARS-CoV-2 shares multiple features with hitherto described coronaviruses, the secondary attack rate (SAR; the number of cases occurring within the incubation period following exposure to a primary case divided by the total susceptible persons (1)) of SARS-CoV-2 appears to be higher (26.3–39.3%, depending on the variant) (2) than infection by SARS-CoV (10.2%) (3) or MERS (4%) (2). The range of clinical signs and symptoms typically showcased by persons infected includes fever, cough, dyspnea, fatigue, and myalgias (4). Among the clinical symptoms reported in persons with coronavirus infection, the prevalence of coughing and its presumptive role in transmission dynamics have attracted attention (5). Although much of the evidence available refers to coughing and the projection of simulated aerosolized viral particles (5), a consensus on airborne transmission was not reached during the initial response to the pandemic (6).

Families and other close social and work contacts have been identified as important contributors towards the pandemic burden (7). Family members and relatives were the main transmission agents of SARS-CoV-2, regardless of whether they shared accommodation with the persons infected (8). However, whilst a systematic review exploring secondary attack rate in different exposure settings highlighted the higher attack rate at homes, social circles, and workplaces (9), most studies focused only on one environment of exposure, such as hospitals or health centers (10–15), households (16, 17), educational centers (18), or public transport (19). Few studies describe the transmission of SARS-CoV-2 related to settings where routine activities such as working, or practicing sports and exercising are carried out, and fewer consider environmental characteristics (ventilation) or factors related to the exposure to the infective agent (exposure time, distancing, etc.).

Wearing a facemask in public settings, maintaining social distance and hand washing are preventive measures and bundles of protective behaviors that had been used for SARS-COV-2 (16, 20–22), and other similar coronaviruses (23). Recent reviews concluded that the use of the face mask is effective in reducing the likelihood of transmission of respiratory pathogens that could include SARS-CoV-2, although most studies included were unrelated to this virus (16, 17). Consequently, we think that is relevant to investigate the effect of wearing a mask specifically in SARS-CoV-2 on the population. In terms of the optimal type of mask, a meta-analysis, found no benefit between different types of masks, with N95 and cloth masks obtaining similar results (17).

In addition to non-pharmacological interventions to address viral transmission, behavioral, social, and environmental factors have also proven vital in the pandemic (24). Contact tracing is an essential and widespread public health measure to mitigate the transmission of infectious diseases (25). During the COVID-19 pandemic, contact tracing was adopted by most countries (26) in addition to other preventive measures such as wearing masks, practicing physical and social distancing, and hand hygiene (27), and was found to be effective in limiting epidemic growth (28). In addition to the operational benefit for clinical and public health management, contact tracing generates a vast amount of data about transmission which could be useful to identify those individuals most at risk of infection (29). Close contact tracing of cases has been one of the main public health approaches to reduce transmission, hospital admissions, and mortality (30). Typically, contact tracing involves interviewing people who are infected to identify which other individuals might have been exposed to the virus, and isolation or quarantine of contact risk (31). Most frequently, the data generated during tracing activities are only used for operational and not research purposes (28).

We aimed to evaluate the association between individual protective measures in the population and SARS-CoV-2 transmission. Also, compare the secondary attack rate of COVID-19 across different settings, exposure, and characteristics.

## Methods

### Study design

An unmatched case–control study selecting the controls from the same population. COVID-19 contact tracing was led by nurses at the COVID-19 Coordinating Centre in Majorca (Balearic Islands, Spain). At the coordination center, all authorized diagnostic testing centers reported every diagnosed case there. Upon receiving this information, nurses contacted the positive patient. During this phone call, the infected patient’s health status was assessed, and close contact tracing was conducted. After concluding the call with the infected patient, close contacts were contacted to notify them of their situation, provide information about the required quarantine, and schedule diagnostic tests as necessary. During the systematic tracing, additional information for this study was collected from the index patient and their close contacts.

During the data collection period, the government of the Balearic Islands imposed mobility restrictions tailored to fluctuating thresholds of coronavirus cumulative incidence, thus allowing, or banning some social activities (32, 33) (both in Table 1). Also, the vaccination rate of the population in the age ranges of our sample was less than 50% (34). During data collection, Mallorca was between 2nd and 4th level of COVID-19 public health measures (“Control Situation” and “High Risk”) (35).

**Table 1:** Balearic Islands’ levels of COVID-19 public health measures.

	Preventive situation (level 0)	Control situation (level 1)	Middle risk (level 2)	High risk (level 3)	Extreme risk (level 4)
Cumulative Incidence**	1–24	25–50	50–150	150–250	>250
Meetings	15 ppl*	10 ppl	6 ppl	6 ppl	0 ppl
Mobility	Without restriction	Lockdown 00 h-06 h	Lockdown 00 h-06 h	Lockdown 00 h-06 h	Lockdown 22 h-06 h
Restaurants	10ppl/table	10ppl/table	6ppl/table	10ppl/table	Close
Ceremonies	75 ppl	50 ppl	30 ppl	20 ppl	15 ppl
Shops	75% occupation	75% occupation	75% occupation	50% occupation	50% occupation
Sports	30 ppl	30 ppl	15 ppl	15 ppl	6 ppl

*People. **New cases in 14 days per 100,000 habitants.

### Definition of index patient, close contacts, cases, and controls

An index patient was defined as a person with a new positive SARS-CoV2 test and unknown origin of their infection during contact tracing.

A ‘close contact’ was a person who (a) had been at the same place as a symptomatic index patient for at least 2 days prior to the onset of symptoms or (b) if the index patient was asymptomatic, at least 2 days prior to the positive diagnosis of the index patient. In addition, in both instances, close contacts should have been within 2 meters of the index patient for more than 15 min within 24 h as per the definition of the Ministry of Health (36).

In our study, cases were defined as those “close contacts” tested positive for SARS-CoV-2 by PCR or antigen test within 10 days after the last contact with the index patient. And controls were similar to cases but had a negative SARS-CoV-2 test result.

### Inclusion and exclusion criteria

Both cases and controls were included in the study if they were older than 18 years and accepted to participate. Cases were excluded if they: (1) were symptomatic close contacts of the index patient, to avoid confusion on the transmission chain. The symptoms considered for exclusion as close contact for this study were the same as those identified as COVID-19 suspicion symptoms by the Spanish Ministry of Health (36); (2) were contacts institutionalized in nursing homes or long-term care facilities, and persons in contact with a healthcare setting (either as workers or patients/service users) -since they had different preventive measures-, and (3) had difficulties with telephone communication or understanding.

### Sample calculation

Accepting an alpha risk of 0.05 and a beta risk of 0.2 in a bilateral contrast, 327 cases and 1,636 controls are required to detect a minimum Odds Ratio of 1.5. It is assumed that the rate of exposure to the different variables collected in the control group will be at least 20%.

Therefore, the total sample will be at least 1,963 close contacts. Considering that each index patient can have an average of about 5 close contacts, about 400 index patients will be necessary.

### Data collection

The questionnaire for the index patients included social-demographic variables (age, sex, education level, profession, and professional status), symptoms and the number of close contacts. The questionnaire for close contacts asked about the environment or setting and exposure characteristics associated with SARS-CoV-2 transmission: contact place, ventilation characteristics (open-air, closed space with or without ventilation), mask-wearing, type of mask, duration of contact, shortest distance, case-contact relationship, household members, and handwashing. Social-demographic variables for close contacts were also collected. Finally, once the isolation period of the close contact ended, test results were retrieved from the electronic health records.

Recruitment of participants was conducted from February to June 2021. During this period, a total of 6,765 patients were reported to the tracking coordination center. We finally included 425 index patients which led to 2,050 close contacts of whom 1,778 were included after application of the inclusion and exclusion criteria.

### Data analysis

Descriptive analysis with sociodemographic variables of index patients and close contacts was performed. Numerical variables were expressed by the mean and standard deviation (SD). Categorical variables were expressed by absolute and relative frequencies. The secondary attack rate with confidence intervals (95% CI) was estimated using the percentage of new cases (positive contacts) among all contacts to enable a comparison with secondary attack rate across the different ventilation characteristics and durations of contact. Logistic regression with random effects adjusted for the index patient was used to calculate Odds Ratios (ORs) and 95% CI to evaluate the association between SARS-CoV-2 infection and all studied factors.

All statistical tests were two-sided, and *p* values <0.05 were considered statistically significant. Statistical analysis was carried out using the Statistical Package for the Social Sciences (SPSS) version 26.0 (IBM SPSS Statistics for Windows, Version 26.0. Armonk, NY: IBM Corp).

### Ethical considerations

The study adhered to the principles of the Declaration of Helsinki and legal regulations regarding data confidentiality and research involving human participants. The study protocol received approval from the Balearic Committee of Clinical Research Ethics (Ref. no: IB 4444/21). All participants were informed of the study’s purpose and procedures before providing their verbal consent to participate.

## Results

Our analysis included all new index patients and their contacts who met inclusion criteria reported to the contact tracing center of Majorca from February to June 2021. During the study period, after doing the contact tracing of all index patients who complied with the inclusion criteria, 2,050 close contacts from 463 index patients were identified. When the inclusion and exclusion criteria were applied, a total of 1,778 close contacts were offered to participate and were accepted (425 cases and 1,353 controls).

### Characteristics of the index patients and contacts

**Figure 1:**
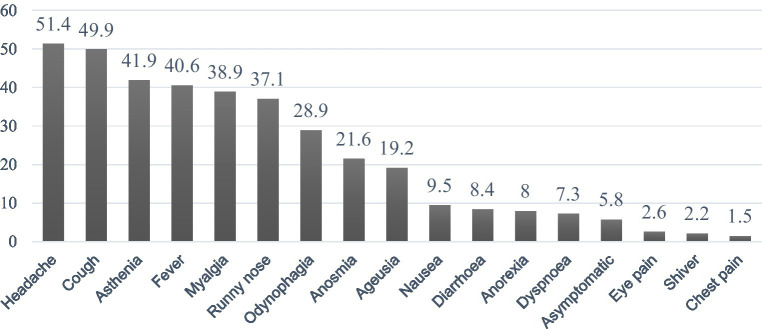
Index patient symptomatology before diagnostic (%). Index patients could have had more than one symptom.

The mean age of index patients was 39.71 ± 15.26, and 53.1% were female. As for education level, 9.4% had not completed basic education or secondary education. Most index patients (59.5%) were office workers (20.5%), worked in the industrial sector (17.4%), or in bar and service workers (11.5%) (Supplementary Table 1). Headaches (51.4%) and cough (49.9%) were the symptoms most frequently reported by index patients, but 6% of them were asymptomatic (Figure 1). Mean close contacts per index patient were 4.43 ± 3.38, including all close contacts identified during the tracing process (*n* = 2,050).

The mean age of the contacts included in the study (*n* = 1,778) was 42.8 ± 17.4, and 53.6% were female (Table 2). Most contacts were family members (57.5), 33.6% shared accommodation, 23.7% were friends, and 13.8% were work colleagues. The accommodation was the main environment of exposure (67.3%). The 60.8% of contacts reported not wearing a mask when they were exposed to the index patient. Finally, the type of mask more frequently used (66.1%) was a surgical one.

**Table 2:** Risk factors associated with SARS-CoV-2 infection among contacts of index patients (*N* = 1,778).

	Control (negative) *N* (%) *N* = 1,353	Case (positive) *N* (%) *N* = 425	OR (95% IC)*	Value of *p*	OR adjusted (95% IC)*	Value of *p*
Sex				0.263		0.617
*M*	635 (46.9)	190 (44.7)				
*F*	718 (53.1)	235 (55.3)	1.11 (0.93–1.32)	0.263	1.06 (0.85–1.32)	0.617
Age group				0.049		0.634
18–27	342 (25.3)	109 (25.6)				
28–42	334 (24.7)	114 (26.8)	1.07 (0.81–1.43)	0.633	1.04 (0.75–1.44)	0.821
43–55	386 (24.1)	115 (27.1)	1.09 (0.82–1.46)	0.559	0.91 (0.64–1.29)	0.609
56–94	351 (25.9)	87 (20.5)	0.73 (0.53–1.00)	0.053	0.87 (0.55–1.21)	0.3
Case-contact relationship				<0.001		0.609
Couples	131 (9.7)	133 (31.3)				
Parents/children/siblings	358 (26.5)	127 (29.9)	0.35 (0.26–0.48)	<0.001	0.72 (0.47–1.10)	0.135
Other relatives	217 (16)	56 (13.2)	0.26 (0.18–0.38)	<0.001	0.84 (0.49–1.44)	0.536
Friendship	350 (25.9)	72 (16.9)	0.21 (0.15–0.30)	<0.001	0.87 (0.49–1.55)	0.644
Work	219 (16.2)	26 (6.1)	0.13 (0.08–0.22)	<0.001	0.64 (0.30–1.38)	0.255
Other	78 (5.8)	11 (2.6)	0.16 (0.06–0.46)	<0.001	0.79 (0.32–1.83)	0.551
Household members				<0.001		
Yes	352 (26.0)	245 (57.6)			–	–
No	1,001 (74.0)	180 (42.4)	0.30 (0.22–0.37)	<0.001		
Exposure settings				<0.001		0.119
Household members	385 (28.5)	209 (49.2)				
Homeplace	479 (35.4)	124 (29.2)	0.52 (0.40–0.71)	<0.001	0.68 (0.50–0.93)	0.018
Leisure	134 (9.9)	29 (6.8)	0.62 (0.41–0.92)	0.018	1.09 (0.64–1.85)	0.768
Workplace	155 (11.5)	33 (7.8)	0.52 (0.34–0.80)	0.003	0.81 (0.48–1.35)	0.415
Sports place	110 (8.1)	16 (3.8)	0.33 (0.15–0.75)	0.008	0.54 (0.26–1.12)	0.098
Education	24 (1.8)	2 (0.5)	0.40 (0.14–1.10)	0.075	0.74 (0.19–2.80)	0.654
Transportation	66 (4.9)	12 (2.8)	0.40 (0.23–0.70)	<0.001	0.55 (0.27–1.11)	0.097
Ventilation characteristics				<0.001		0.004
Closed without ventilation	415 (30.7)	243 (57.2)			0.75 (0.55–1.03)	
Closed with ventilation	585 (43.2)	152 (35.8)	0.45 (0.34–0.60)	<0.001	0.43 (0.26–0.71)	0.072
Open-air	353 (26.1)	30 (7.1)	0.17 (0.11–0.26)	<0.001		<0.001
Wearing mask				<0.001		0.029
No	734 (54.2)	347 (81.6)				
Sometimes	347 (25.69)	54 (12.7)	0.36 (0.25–0.50)	<0.001	0.70 (0.45–1.07)	0.099
Yes	272 (20.1)	24 (5.6)	0.20 (0.13–0.32)	<0.001	0.47 (0.26–0.82)	0.010
Type of mask				0.770		
Not wearing mask	1,112 (82.2)	370 (87.1)				
Nonmedical mask	23 (1.7)	6 (1.4)	1.031 (0.52–2.03)	0.930	–	–
Medical mask	161 (11.9)	34 (8)	0.82 (0.57–1.20)	0.298		
FFP2 mask	57 (4.2)	15 (3.5)	0.95 (0.56–1.61)	0.844		
Exposure time	*N* = 1,353	*N* = 424		<0.001		<0.001
>24 h	181 (13.4)	186 (43.9)				
4–24 h	244 (18)	105 (24.8)	0.41 (0.30–0.57)	<0.001	0.55 (0.37–0.81)	0.003
1–4 h	463 (34.2)	95 (22.4)	0.21 (0.16–0.30)	<0.001	0.39 (0.26–0.60)	<0.001
20 min – 1 h	368 (27.2)	29 (6.8)	0.08 (0.06–0.12)	<0.001	0.19 (0.11–0.33)	<0.001
15–20 min	97 (7.2)	9 (2.1)	0.11 (0.05–0.29)	<0.001	0.25 (0.11–0.56)	<0.001
Shortest distance	*N* = 1,352	*N* = 424		<0.001		0.054
0 m	297 (22)	194 (45.8)				
0–1 m	632 (46.7)	186 (43.9)	0.42 (0.32–0.55)	<0.001	1.07 (0.71–1.61)	0.758
1–2 m	423 (31.3)	44 (10.4)	0.16 (0.11–0.24)	<0.001	0.64 (0.38–1.10)	0.109
Handwashing				0.96		0.599
1 time a day	12 (0.9)	3 (0.7)				
2–3 times a day	162 (12)	51 (12)	1.15 (0.32–4.16)	0.83	1.04 (0.21–5.14)	0.961
> 3 times a day	1,179 (87.1)	371 (87.3)	1.10 (0.30–4.02)	0.88	0.85 (0.17–4.18)	0.840
Cough				0.012		0.007
No	694 (51.5)	180 (42.7)				
Yes	653 (48.5)	242 (57.3)	1.47 (1.08–1.95)	0.012	1.57 (1.13–2.20)	0.007
Fever						0.424
No	869 (64.5)	238 (56.4)				
Yes	478 (35.5)	184 (43.6)	1.29 (0.96–1.73)	0.090	1.15 (0.82–1.60)	0.424

*Crude and adjusted odds ratios were estimated using random-effects logistic regressions adjusted for the index patient.

The secondary attack rate within the contacts was 24.0% (95% CI: 22.0–26.0%). According to ventilation characteristics, was: 7.8% (95% CI: 5.1–10.5%) in open-air, 20.6% (95% CI: 17.7–23.5%) in closed space with ventilation, and 36.9% (95% CI: 33.2–40.6%) in unventilated closed spaces. By exposure time, was: 8.5% (95% CI: 3.2–13.8%) for 15–20 min, 7.3% (95% CI: 4.7–9.9%) for 20 min–1 h, 17.0% (95% CI: 14.0–20.1%) 1–4 h, 30.1% (95% CI: 25.3–34.9%) for 4 h-24 h, and 50.7% (95% CI: 45.6–55.8%) for close contacts who stayed more than 24 h. Regarding the secondary attack rate in different exposure settings: among household members, it was 35.2% (95% CI: 31.3–39.0%), at home was 20.6% (95% CI: 17.3–23.8%), social settings 17.8% (95% CI: 11.9–23.7%), and finally, the work setting 17.6% (95% CI: 12.1–23.0%) (Table 3).

**Table 3:** Secondary attack rate within the contacts identified through tracing.

	SAR	
	*n*	% CI 95%
Overall	425/ 1778	24.0 (22.0–26.0)
Ventilation		
Open-air	30/383	7.8 (5.1–10.5)
Closed space ventilated	152/737	20.6 (17.7–23.5)
Closed space without ventilation	243/658	36.9 (33.2–40.6)
Exposure time		
15 min–20 min	9/106	8.5 (3.2–13.8)
>20 min – 1 h	29/397	7.3 (4.7–9.9)
1–4 h	95/558	17.0 (14.0–20.1)
4–24 h	105/349	30.1 (25.3–34.9)
>24 h	186/367	50.7 (45.6–55.8)
Exposure settings		
Household members	209/594	35.2 (31.3–39.0)
Household	124/603	20.6 (17.3–23.8)
Leisure	29/163	17.8 (11.9–23.7)
Workplace	33/188	17.6 (12.1–23.0)
Sports place	16/126	12.6 (6.9–18.5)
Education	2/26	7.7 (2.6–17.9)
Transportation	12/78	17.1 (7.4–23.4)

### Effects of personal protective measures and exposure on transmission

The bivariate analyses showed an inverse association between personal protective measures and exposure characteristics (exposure time and ventilation) and SARS-CoV-2 transmission. There were no differences between sex and infection of either index patients or close contacts who were subsequently infected. Among the close contacts, there was a close statistically significant difference for age group and SARS-CoV-2 transmission; OR 1.07 (95% CI: 0.81–1.1.43) between 28 and 42 years, 1.09 (95%CI: 0.82–1.46) for 43–55 years and 0.73 (95% CI: 0.53–1.00) for 56–94 years compared with 18–27 age group.

Contacts who shared accommodation with the index patient were more likely to be infected (OR for non-household members 0.30, 95% CI: 0.22–0.37). Similarly, when the exposure environment was analyzed, close contacts in homes, workplaces, and sites of leisure and socialization activities (bars and restaurants) were more likely to be infected compared to those who were exposed to transportation, education, and sports environments (*p* < 0.001).

Close contacts who were exposed in the open air were less likely to be infected (OR 0.17, 95% CI: 0.11–0.26) than those exposed to index patients in unventilated, closed spaces. Those contacts who maintained a physical distance between 1 and 2 meters were less likely to be infected (OR 0.16, 95% CI: 0.11–0.24) than close contacts who were closer than 1 m. Infection was less likely to occur among those spending less than 1 h with the index patient, compared to those close contacts who had spent more than 24 h (OR 0.08; 95% CI: 0.06–0.12). Cough and fever of the index patient were more likely to result in infection of the close contacts identified; OR 1.47; 95% CI: 1.08–1.95 and 1.29; 95% CI: 0.96–1.73, respectively. We did not find a statistically significant association between a particular type of face mask and infection, but wearing any mask was however associated with less risk of infection (*p* < 0.001) (Table 2).

In a multivariant analysis, adjusted odds ratio analyses showed that open-air setting (OR 0.43, 95% CI: 0.26–0.71), exposure for less than 1 h (OR 0.19, 95% CI: 0.11–0.33), and wearing a mask (OR 0.47, 95% CI: 0.26–0.82) had a protective effect against infection. Also, if the cough was present in index patients the likelihood of transmission to their close contacts increased (OR 1.57, 95% CI: 1.13–2.20). The multivariable analyses did not include the type of mask and those household members because of collinearity with wearing masks and relationships with index patient-contact, respectively (Table 2).

## Discussion

Our results characterized the transmission of COVID-19 in the community. Wearing face masks was effective in reducing this transmission, regardless of the type of masks used. Additional factors such as duration of exposure to a person with infection, and environmental ventilation, were also influential in community transmission, rather than the relationship with the index patient and the areas where exposure occurred. Our results emphasize the contribution of households and the sharing of accommodation towards the transmission dynamics of the pandemic. In this regard, we observed that limiting the time of exposure, increasing ventilation, and wearing any type of mask as much as possible would prevent SARS-CoV-2 transmission.

In our study, we did not observe a statistically significant difference regarding sex in the transmission of SARS-CoV-2 to close contacts. This finding is aligned with other already published studies (18). Similarly, we found no statistical differences regarding the age of contacts, whereas higher rates of infection among close contacts who were older have been reported (22, 37, 38). However, these studies were mainly conducted in 2020, when social awareness of compliance with personal protective measures to reduce the risk of infection was heightened (39). Our results could be explained by prevailing political narratives and directives towards protecting older adults, messages which were readily adopted by the population (40).

In our study, the overall secondary attack rate was ~24%, a slightly higher percentage, but similar to results typically conducted in households, ranging between 16.5 and 23.0% (22, 37, 41–43). However, environments such as boxing venues and nightclubs seem to have a higher secondary attack rate than households, whilst the workplace was associated with the lowest (42). Another community study focused on schools reported 9.2% secondary attack rate, slightly higher than our results (7.7%) (37). Our results provide evidence that the household was the environment with the higher risk of secondary attack rate within all daily settings. Similar findings have been obtained elsewhere (44, 45); household secondary attack rate was higher than global, followed by social (44).

Differences were found when the secondary attack rate was calculated for different exposure times and ventilation characteristics. Compared to outdoor spaces, secondary attack rate in closed unventilated spaces was almost 5 times higher. A study that assessed poor ventilation in a restaurant concluded that indeed ventilation in indoor spaces at restaurants had a lower risk than spaces without ventilation (46). Exposure time is a relevant variable in transmission; spending more than 4 h with an infected person has been observed to double the transmission rate. Our results were higher than those reported elsewhere for a time exposure of more than 60 min with a secondary attack rate of 24% (42). Contacts who spent more than 24 h in closed unventilated spaces tended to be household members, therefore, our results reinforce the idea that much of the growth in community transmission during the pandemic can be attributed to the household and close relatives (18).

We observed a higher risk of transmission among close contacts when the index patient had a cough. Previous evidence reflected simulated conditions (5, 47). The smaller cough droplets can reach longer distances (47), and sneezing projects more viral load than coughing (5). Our study showed similar results to *in vitro* studies who observed increased shedding of droplets containing SARs-CoV-2 when coughing, coinciding with increased transmission and infection of close contacts in the general population.

Mask-wearing could be one of the most important individual behaviors to reduce SARS-CoV-2 transmission, compared with other personal protective variables (hand hygiene washing and social distance). Other studies focused on SARS-CoV-2 were consistent with our results. Wearing a mask at home after illness onset reduces the transmission of SARS-CoV-2 (OR 0.30, 95% CI: 0.11–0.82) compared with people who never wear a mask (22).

The type of mask (cloth, surgical, FFP2) used during the exposure did not show statistically significant differences in the risk of transmission (42). Existing evidence suggests a gradient of protection, with N95 respirator use associated with viral infectious episodes for healthcare workers compared with surgical masks (48). Such reduction could be attributed to the higher risk of infection in a clinical setting compared to other social environments (49). In another study, masks indicated less effectiveness but still concludes that any type of mask minimizes the risk of transmission in the general population (50). Therefore, the type of mask perhaps will not be as important in the community as in health centers.

Household members accounted for more than half of all at-risk contacts who became infected, making the home the environment with the highest risk for SARS-CoV-2 transmission. A cohort study that compared households with other settings got similar results (45). In a multivariable model, household members had an OR of 8.1 (95% CI: 5.9–11.4) compared with shared transportation, enclosed space without direct contact, and conversation (45). Few studies analyzed all areas at the same time, which makes it difficult to compare the obtained results with existing ones.

### Limitations

Our study has some limitations due to the design and methods followed, which should encourage caution when interpreting the results. First, case–control studies may overestimate the magnitude of the effect seen when compared to relative risk (51). Secondly, we considered that each positive contact had been infected by an index patient, whereas it might be possible for some contacts to be infected by other individuals in the community unknown to the contact tracing team. Another limitation could be the exclusion of children could have led to an underestimated secondary attack rate in the educational environment. We understand that this population requires attention but due to the tracing logistics, we opted to exclude them from the study. Also, the contact tracing interview focused on the use of face masks but did not clarify whether such use was correct (i.e., mouth and nose covered), or whether the face mask was new or worn out, both factors which can compromise the efficacy of the mask (52). As well as the limitation of not to collect the vaccination status of our participants. Finally, we only asked participants about the symptoms included in the national protocol developed by the Ministry and did not ask about other symptoms experienced by the index patients such as sneezing, which could be associated with transmission independently from cough (47). Finally, selection bias for the close contacts could have affected this study because the index patients voluntarily named their close contacts, being likely to not have included every possible close contact.

Regarding the study strengths, most index patients diagnosed from February to June 2021 were included in the study. We included all COVID tests realized in Majorca during the period of study so the results could be generalized to the entire community. We designed the study to minimize the biases of case–control studies (complacency bias, and recall bias) (53). Complacency bias was minimized as the questionnaire was administered before the respondents were given the result of their test, avoiding any influence on their responses. Recall bias was minimized by contacting people at the time of their identification as close contacts, thus minimizing the time elapsed between the call and the day of exposure.

## Conclusion

Our results provide evidence supporting individual and collective prevention and safety measures against coronavirus transmission. Understanding the association between the use of masks, ventilation of area, time exposure, and the transmission of SARS-CoV-2 could optimize the real-time contact tracing mechanisms and focus on the more at-risk population. Also, additional attention and resources could be allocated to close contacts of index patients with cough, which would be at higher risk of infection.

Our study may have been beneficial in assisting public institutions to better identify individuals most at risk, thereby optimizing the use of limited human resources in case tracing. Despite organizational difficulties and pressure on the health system and workforce during contact tracing, research opportunities were afforded to understand community mechanisms of transmission and thus improve this public health component. Studies replicating our experience should be embedded in contact tracing efforts, even during pandemic events, to refine policy or tracing decisions.

## Data availability statement

The dataset supporting the findings of this study is available in the Docsalut repository at https://hdl.handle.net/20.500.13003/20064, doi: 10.52207/docusalut.20064.

## Ethics statement

The studies involving humans were approved by Balearic Ethical Committee of Clinical Research (Ref. No: IB 4444/21). The studies were conducted in accordance with the local legislation and institutional requirements. The participants provided their written informed consent to participate in this study.

## Author contributions

AH-T: Conceptualization, Data curation, Formal analysis, Investigation, Writing – original draft. EC-S: Formal analysis, Supervision, Writing – review & editing. LC-M: Data curation, Formal analysis, Writing – review & editing. CS-R: Data curation, Validation, Writing – review & editing. MB-V: Conceptualization, Formal analysis, Methodology, Validation, Writing – review & editing. AY: Conceptualization, Formal analysis, Validation, Writing – review & editing.
