# Supplementary material for: Personal protective measures and settings on the risk of SARS-COV-2 community transmission: a case–control study
Source: Front Public Health. 2024 Jan 8;11:1327082. doi: 10.3389/fpubh.2023.1327082 (PMC10801386; doi:10.3389/fpubh.2023.1327082)
Supplement: Supplementary file 1 [file Table_1.DOCX]

| Supplementary table. Descriptive characteristics of the index patient. N=463 | |
| --- | --- |
| Age mean (SD)* | 39.71 (15.3) |
| Sex  Male  Female | 246 (53.1)  217 (46.9) |
| Level study  Elementary School  High School  High School degree  Professional Training  University Study | N= 457  43 (9.4)  106 (23.1)  71 (15.5)  102 (22.2)  135 (29.4) |
| Profession  Catering industry  Sociosanitary professional  Sanitary professional  Industry sector  Office work  Informal care  Teacher  Student  Cleaning staff  Coach driver  Tourism sector  Others | N=419  48 (11.5)  8 (1.9)  9 (2.1)  73 (17.4)  86 (20.5)  13 (3.1)  23 (5.5)  37 (8.8)  13 (3.1)  7 (1.7)  13 (3.1)  89 (21.3) |
| Employment status  Work stoppage  Active  Retired | N=435  145 (33.3)  259 (59.5)  31 (7.1) |
| Contacts per index patient  mean (SD)* | 4.43 (3.38) |

Results are reported as mean ± SD or n (%). *Mean contacts per index patient included all the contacts of the tracing, including contacts who couldn’t participate (n=2050).
